# Perspectives of the COVID-19 Pandemic on Reddit: Comparative Natural Language Processing Study of the United States, the United Kingdom, Canada, and Australia

**DOI:** 10.2196/36941

**Published:** 2022-09-27

**Authors:** Mengke Hu, Mike Conway

**Affiliations:** 1 Department of Biomedical Informatics University of Utah Salt Lake City, UT United States; 2 School of Computing & Information Systems University of Melbourne Carlton Australia; 3 Centre for Digital Transformation of Health University of Melbourne Carlton Australia

**Keywords:** COVID-19, social media, natural language processing, Reddit

## Abstract

**Background:**

Since COVID-19 was declared a pandemic by the World Health Organization on March 11, 2020, the disease has had an unprecedented impact worldwide. Social media such as Reddit can serve as a resource for enhancing situational awareness, particularly regarding monitoring public attitudes and behavior during the crisis. Insights gained can then be utilized to better understand public attitudes and behaviors during the COVID-19 crisis, and to support communication and health-promotion messaging.

**Objective:**

The aim of this study was to compare public attitudes toward the 2020-2021 COVID-19 pandemic across four predominantly English-speaking countries (the United States, the United Kingdom, Canada, and Australia) using data derived from the social media platform Reddit.

**Methods:**

We utilized a topic modeling natural language processing method (more specifically latent Dirichlet allocation). Topic modeling is a popular unsupervised learning technique that can be used to automatically infer topics (ie, semantically related categories) from a large corpus of text. We derived our data from six country-specific, COVID-19–related subreddits (r/CoronavirusAustralia, r/CoronavirusDownunder, r/CoronavirusCanada, r/CanadaCoronavirus, r/CoronavirusUK, and r/coronavirusus). We used topic modeling methods to investigate and compare topics of concern for each country.

**Results:**

Our consolidated Reddit data set consisted of 84,229 initiating posts and 1,094,853 associated comments collected between February and November 2020 for the United States, the United Kingdom, Canada, and Australia. The volume of posting in COVID-19–related subreddits declined consistently across all four countries during the study period (February 2020 to November 2020). During lockdown events, the volume of posts peaked. The UK and Australian subreddits contained much more evidence-based policy discussion than the US or Canadian subreddits.

**Conclusions:**

This study provides evidence to support the contention that there are key differences between salient topics discussed across the four countries on the Reddit platform. Further, our approach indicates that Reddit data have the potential to provide insights not readily apparent in survey-based approaches.

## Introduction

In December 2019, several cases of respiratory disease were reported in Wuhan City, China [1]. This respiratory disease, ultimately named COVID-19, was caused by a novel coronavirus identified as SARS-CoV-2. COVID-19 is a highly contagious infection, typically spread through respiratory droplets or by contact [2]. In the period since COVID-19 was declared a pandemic by the World Health Organization (WHO) on March 11, 2020, the disease has had an unprecedented impact worldwide, with, as of June 13, 2022, more than 540 million confirmed cases and 6.3 million deaths [3]. The number of people who have died because of the COVID-19 pandemic could be roughly three times higher than official figures suggest, according to a new analysis [4].

To suppress the transmission of COVID-19, governments have enforced several waves of border shutdowns, travel restrictions, quarantine, and other nonpharmaceutical interventions such as mask mandates, limiting public activities, and restricting travel [5-7], sparking fears of social unrest, educational disruption, and economic crisis [8]. The scientific uncertainties regarding the virus and its transmission have created a volatile political and social environment [9,10]. These concerns are exacerbated by the dynamic nature of the virus, with new variants emerging over time [10,11], creating uncertainty regarding the projected course of the pandemic and impacts on policy. Further, the advent of COVID-19 has been associated with a marked deterioration in population-level mental health issues, especially for vulnerable populations such as college students and pregnant women [12-14].

Traditional surveillance systems, including those utilized by the US Centers for Disease Control and Prevention and the European Influenza Surveillance Scheme, rely on both virologic and clinical data, and publish data once per week, typically with a 1-2–week reporting lag [15]. Survey data have also been leveraged to investigate the spread of COVID-19 in the community. In particular, ecological momentary assessment has proven to be a valuable research tool [16]. Further, the peer-reviewed scientific literature and preprint data are popular data sources to study the impact of COVID-19.

Social media such as Reddit, Twitter, Facebook, Weibo, and others provide a readily available source of abundant, organic, publicly accessible first-person narratives [17-25], which can serve as data sets for identifying outbreaks and providing situational awareness. Even more important during the COVID-19 pandemic, social media data provide a means of better understanding public attitudes and behaviors during a crisis to support communication and health-promotion messaging, especially in situations in which survey data are not readily available [15,26].

During lockdown events, social media platforms have—through their individual users—provided informational support and online access to services for pregnant women to obtain prenatal care services, such as consulting and scheduling necessary appointments [27]. Similarly, Weibo posts have proved useful in investigating public attitudes toward COVID-19 vaccination in China [28,29]. Alternative data sources such as Reddit are especially valuable in situations where traditional survey data are limited. For example, Reddit has been employed to study the impact of the pandemic on disordered eating behaviors [30].

Topic modeling, a popular statistical unsupervised machine-learning technique, has been widely used for discovering the underlying themes that occur in collections of health-related texts [31]. Because of its utility in facilitating the analysis of large-scale document collections, useful results have been obtained in areas such as biological/biomedical text mining; clinical informatics; and information extraction from other text data sources, including government reports, newspaper articles, and scientific journals [32]. Social media data such as Reddit are frequently used in conjunction with topic modeling methods to explore public concerns, attitudes, and policies. For example, Zhang et al [33] identified eight popular topics using Chinese social media platforms that served to characterize the COVID-19 infodemic, including conspiracy theories, government response, preventive action, new cases, transmission routes, origin and nomenclature, vaccines and medicines, and symptoms and detection. Topic modeling has also been used to examine COVID-19–related concerns across different countries [34]. Categorizing posts by topic modeling technique such as latent Dirichlet allocation (LDA) [35], perhaps the most popular topic modeling method, has been used extensively to analyze sentiments and concerns during the COVID-19 crisis [1,10,19,20,36-41], especially in the context of large social media data sets. Topic modeling with LDA has also demonstrated utility in discovering themes from combined data sets, such as combining news articles and tweets in Brazil to study the impact of COVID-19 [42]. LDA has also been used to study sentiment variations over time [10,43-46]. In particular, as COVID-19 vaccine–related issues received increasing public attention, LDA was employed to study the changes in people’s opinions toward COVID-19 vaccination, discovering that public attitudes became more favorable over time [47,48]. However, whether the topics identified are interpretable typically requires qualitative evaluation [49,50].

Reddit is one of the most popular social media platforms with over 430 million active users and 1.2 million subreddits (ie, topic-focused subforums) as of May 2020, with over 70% of its user base coming from English-speaking countries [51,52]. Some subreddits have clear descriptions regarding locations (eg, *r/CoronavirusUK, r/CanadaCoronavirus*), which enables a more targeted analysis of users from different countries [43].

In this work, we employed Reddit data from six geographically specific COVID-19–related subreddits representing four English-speaking countries, the United States, the United Kingdom, Canada, and Australia, to investigate (1) whether there were key differences between salient topics discussed across the four countries and (2) whether Reddit data have the potential to provide insights not readily apparent in survey-based approaches. In general, LDA topic modeling was applied to each country-specific Reddit data set. We trained multiple topic models for each country consisting of a different number of topics and manually inspected each model to find the optimal model for each country (ie, the model that generated the most coherent and least redundant topics). We further compared the summarized topics for each country based on each country’s model, and mapped them to four common topic categories (ie, metacategories). Finally, longitudinal topic trends were examined to identify trends in the common topic categories, which were then mapped to the COVID-19 events for each country.

## Methods

### Data Collection and Preprocessing

As Reddit data do not generally include geolocation information, we collected data from the six most popular subreddits (topical forums on Reddit) related to the United States, the United Kingdom, Canada, and Australia (*r/CoronavirusUK, r/coronavirusus, r/CoronavirusCanada, r/CanadaCoronavirus, r/CoronavirusAustralia, r/CoronavirusDownunder*), as shown in Table 1.

Data were collected using the pushshift.io [52] application programming interface (API), a service that archives Reddit data to its online database in real time. We employed the pushshift.io API to harvest COVID-19–related data, as previous work has indicated that this approach yields a more complete data set than alternative methods (eg, the PRAW API) [53]. However, in the data collection process, we noticed that pushshift.io failed to identify all of the new updates, including deleted comments [54]. To ensure we collected the most complete data set possible, we recollected the data over the same time frame after 3 months and consolidated the new and old data sets to gain a more complete data set.

The consolidated Reddit data set consisted of 84,229 initiating posts and 1,094,853 associated comments collected between February and November 2020 derived from the six subreddits shown in Table 1. These subreddits are related to a specific country according to the subreddit description. For example, *r/CanadaCoronavirus* is used primarily by Canadians to discuss the COVID-19 crisis. Among all the country-specific COVID-19 subreddits, the six subreddits we chose have the largest number of members (>8000), which means they are the most active and popular geographically specific COVID-19–related subreddits available. As users typically present their own experiences in the initiating posts [55], with subsequent comments frequently subject to off-topic discussion, we restricted our topic study to only initiating posts. Given that Reddit does not provide user-level geolocation information, we regarded the fact that a Reddit user posted in a country-specific subreddit as a proxy for their location in that country.

To build the corpus for each country, we organized the submissions from the six subreddits shown in Table 1. For example, to build an Australia data set, we extracted all text data (the title section and the description section) from the submissions of *r/CoronavirusAustralia* and *r/CoronavirusDownunder*. We then automatically identified URLs and email addresses, which were removed from the texts of submissions to simplify the subsequent topic modeling process. To remove the stop words (ie, common English function words such as “the,” “of,” and “it”), we first used the Natural Language Toolkit (NLTK 3.3 for Python 2.7) [56] to initialize the stop-words list. The stop-words list was then further augmented using the Essential Word List (a lexicon originally developed for language learning and testing) [57]. Subsequently, the text data from submissions were tokenized (ie, the string *Let’s go!* was tokenized into the list “let,” “’s,” “go,” “!”) and lemmatized (ie, the string *I was reading the paper* was broken down into the list “I,” “be,” “read,” “the,” “paper”) using the Python SpaCy 2.2.1 package [58] to convert various forms of words (eg, *cough, coughing*) into a canonical form (eg, *cough*).

**Table 1:** Subreddit information on July 31, 2021.

Country	Subreddit	Number of members	Date subreddit created
United Kingdom	*r/CoronavirusUK*	92,600	February 11, 2020
United States	*r/coronavirusus*	141,000	February 12, 2020
Canada	*r/CoronavirusCanada*	9000	February 12, 2020
Canada	*r/CanadaCoronavirus*	67,300	March 1, 2020
Australia	*r/CoronavirusAustralia*	10,800	February 21, 2020
Australia	*r/CoronavirusDownunder*	90,300	February 23, 2020

### Topic Modeling and Common Topic Annotation

**Figure 1:**
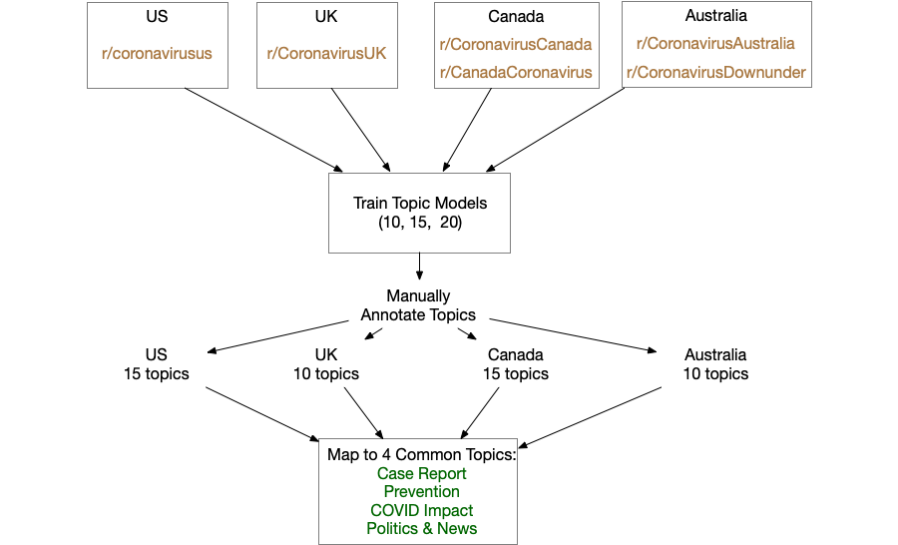
Procedure for topic training and mapping to the common topic categories.

We used the topic modeling technique to compare the broad themes emerging from the United States, the United Kingdom, Canada, and Australia. The general procedure is described in Figure 1. Specifically, we adopted a generative probabilistic modeling algorithm, LDA, which models documents as random mixtures over topics, where each topic is characterized as a distribution of words [35].

We trained multiple topic models (consisting of 10, 15, and 20 topics) for each of the four countries using the LDA implementation in the Gensim 3.8.3 [59] toolkit. Under each model, we summarized the topics according to the topic keywords. We then manually checked if the topics overlapped or were redundant. We found that topics thematically overlapped when the model contained fewer than 10 topics, while the topics were redundant when the model had more than 20 topics. Thus, we chose 10, 15, and 20 topics to train the models for further manual examination.

For each topic model, the most characteristic keywords associated with each of the thematic topics were manually examined, focusing specifically on the posts that were particularly representative according to the contribution probability of those topics to determine which model best characterized the data set. In the process of manual identification of topics, we noticed that the models for the four countries had different optimal numbers of coherent, nonoverlapping topics. Further, some models contained topics idiosyncratic to that country (ie, they did not appear in the models of other countries). For example, the “mental health” topic in the UK topic model did not appear in the US topic model. To compare and contrast the common themes among the four countries, we consolidated these various topics into four common topic categories. Topics and their mappings to the common topic categories are listed in Table 2.

**Table 2:** Consolidated topics common across all four countries.

Common topic	Topics
COVID Impact	work, finance, education, travel restriction, social distancing
COVID Prevention	mask wearing, hand washing, transmission risk
Case Report	case report, report of interaction with hospital
Policy & News	policy announcement, news, question and answer

### Common Topic Prevalence in the United States, the United Kingdom, Canada, and Australia

The prevalence of common topics for the United States, the United Kingdom, Canada, and Australia was studied by first finding the “document-topic” for each post. The document-topic refers to a topic that is the major constituent (according to the contribution probability) of a given document [60], which can be used to study the proportion of a specific topic for each country-related data set. As the topics and their distributions vary among the US, UK, Canada, and Australia data sets, the document-topics were analyzed separately based on each country-related data set. To find document-topics for each country, we needed to first find the threshold probability to identify the major topics. Specifically, for each country-related data set, if the topic probability for a certain document was above the threshold, this topic was deemed to be one of the major constituents for this document. Practically, document-topics were not uniformly distributed (ie, some documents contain more than one while some contain no document-topic). To evenly address each country-related data set, we iteratively tested different candidate probability values until the number of document-topics was close to the number of documents in that country-related data set. More precisely, from the topic models we trained for each country, we have: (1) a set of topics, (2) a list of words (we used 40 words) associated with each topic ranked by their contribution probability to that topic, and (3) a list of documents (submission posts) with estimates of the proportion of each topic. To find the threshold, whenever we set up the threshold probability for testing, we counted the number of document-topics for each submission and summed them for all submissions until the total number of document-topics was close to the number of submissions in that country-related data set. The reason for carrying out this process was to help ensure that the document-topics accurately covered the topics of all submissions in the Reddit data set, thus maximizing the proportion of content that was represented [60]. We repeated this process until finding the threshold for each country.

Using the document-topic threshold for each country, we identified the proportion of each topic by first calculating the number of posts whose topic probability was above the threshold, and then dividing this number by the total number of posts to obtain the topic proportion. The proportion of the common topic categories was determined by summing the proportion of the topics that belonged to each common topics category.

### Common Topic Trend in Reddit and COVID-19 Event Timeline

With the document-topic threshold for each country, we also calculated the number of submissions on a specific common topic category for each week, before counting the weekly volume of submissions on each common topic category, to plot the common topic trend for each country from February to November in 2020. We also mapped the COVID-19 event timeline from the WHO [61] and Think Global Health [62] to our Reddit data trend plot for comparison.

### Ethical Considerations

We restricted our analysis to publicly available discussion content and the University of Utah’s Institutional Review Board exempted the study procedure and data from ethical review (IRB_00076188) under Exemption 2 as defined in the United States' Code of Federal Regulations (CFR), 45 CFR 46.101(b).

## Results

### Corpus Characteristics

Our COVID-19 Reddit data set comprises 10 months of discussions (February 2020 to November 2020), which covers the main early COVID-19 events, including the initial outbreak and subsequent lockdowns in the United States, the United Kingdom, Canada, and Australia. During this time, 103,180 unique users posted some 84,229 submissions and their associated 1,094,853 comments. Table 3 summarizes the numbers of unique users, submissions, and associated comments for each subreddit.

**Figure 2:**
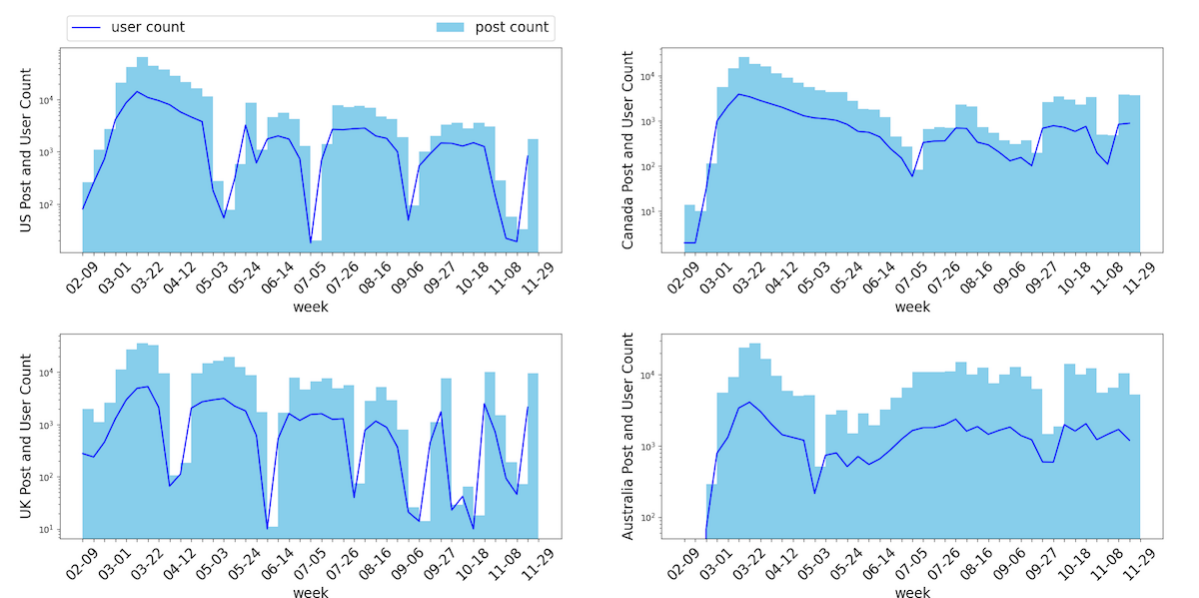
Post and user volume by week (log scale).

To further study the behavior of posting on Reddit, we summarized the weekly post volume and user volume for each country, as shown in Figure 2. We found that the user volume is consistent with the post volume, which indicates that posts are created by organic Reddit users rather than by a “water army” [63] of paid posters. Thus, the post data we used for analysis can be considered to be reflective of subreddit users’ genuine opinions and behavior during the COVID-19 crisis. We also noticed that for the four countries, the highest volume peak appeared between February and April 2020 when the first wave of lockdowns were enforced. Moreover, the post volume and user volume decreased over time.

**Table 3:** Reddit COVID-19 data from February 2020 to November 2020.

Country data set	Subreddit	Unique users, n	Submissions, n
United Kingdom	r/CoronavirusUK	20,482	17,350
United States	r/coronavirusus	55,380	35,885
Canada	r/CoronavirusCanada	4061	4625
Canada	r/CanadaCoronavirus	10,420	9670
Australia	r/CoronavirusAustralia	3114	2359
Australia	r/CoronavirusDownunder	15,537	14,340

### Results from Topic Modeling With Common Topic Annotation

After manually examining the topic models (10, 15, 20 topics) for the United States, the United Kingdom, Canada, and Australia, we qualitatively identified the most coherent model, as well as the threshold of the document-topics for each country-related data set, as shown in Table 4. The reason we chose the model manually instead of using automated methods (eg, LDA coherent score) is due to the limitation of topic model interpretation [49].

**Table 4:** Manually selected topic models for the United States, the United Kingdom, Canada, and Australia, and the associated document-topic thresholds.

Country	Chosen model	Threshold for document-topics
United States	15-topics model	0.19881
United Kingdom	10-topics mode	0.24
Canada	15-topics model	0.15864215
Australia	10-topics model	0.18434

### Common Topic Prevalence in the United States, the United Kingdom, Canada, and Australia

**Figure 3:**
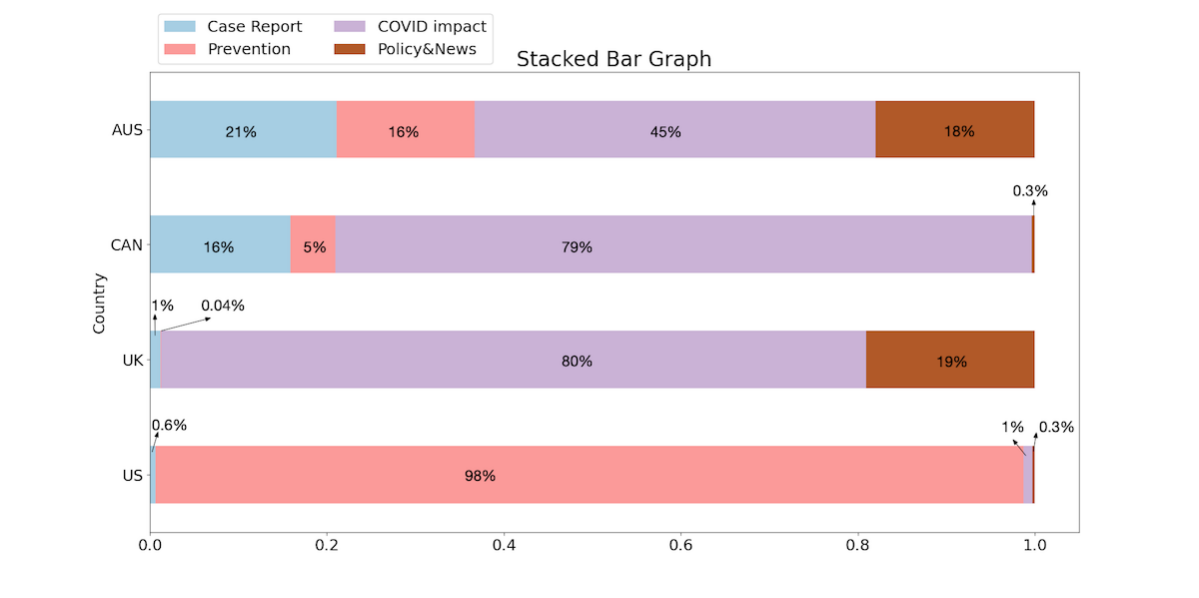
Topic proportions for the United States (US), the United Kingdom (UK), Canada (CAN), and Australia (AUS).

For each topic in each model, we mapped that topic to four common topics (described in Table 2) and calculated the number of documents for each topic according to the thresholds shown in Table 4. The document proportion for each topic for each country is presented in Figure 3. The detailed calculations for generating Figure 3 are presented in Multimedia Appendix 1.

We found that the majority of the US posts focused on COVID-19 prevention strategies, whereas the posts in the United Kingdom, Canada, and Australia were more focused on the impacts of COVID-19, including education, finance, and potentially limited availability of food.

### Common Topic Trend in Reddit and COVID-19 Event Timeline

**Figure 4:**
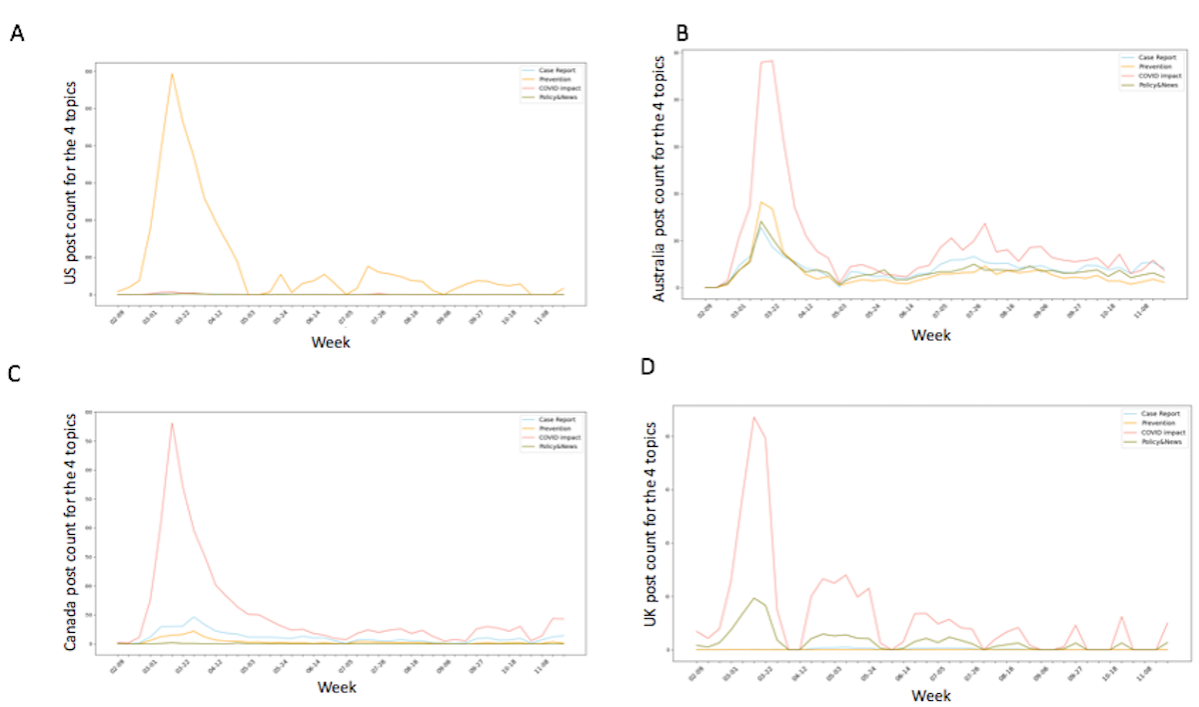
Topic weekly trend for (A) the United States, (B) Australia, (C) Canada, and (D) the United Kingdom. Higher-resolution version of this figure is available in Multimedia Appendix 2.

In visualizing the identified topic model, we also summarize the topic trends for the United States, the United Kingdom, Canada, and Australia in Figure 4.

In both Figure 2 and Figure 4, it can be observed that all countries experienced an early peak in posting activity. The user volume plot in Figure 2 and trends in Figure 4 imply that users post more during lockdown events. For all four countries, the post volume and user volume reached a peak in March 2020. In the same month, all of these countries announced lockdown or travel restriction policies. This increase in posts may reflect a combination of public fear and concern regarding the virus, and the fact that many individuals found themselves confined to their homes, with abundant time to access social media. A list of salient pandemic-related events is shown in Table 5.

**Table 5:** Timeline of COVID-19 lockdown events in 2020 [62].

Date	Event
March 11	United Kingdom lockdown; United States announces level 3 travel advisory
March 18	United States and Canada suspend nonessential travel between the two countries
March 23	United Kingdom lockdown
March 24	Australia bans all overseas travel
April 18	United States: protests of the country’s lockdown
June 24	United States: increase in case rates in 26 states since easing lockdown restrictions
July 3	United Kingdom announces an end to travel restrictions except for the United States
July 4	Melbourne, Australia tightens restrictions on 12 suburbs
September 5	Australia extends its hard lockdown until the end of September
October 12	United Kingdom announces new lockdown rules

## Discussion

### Principal Findings

In this work, we applied topic modeling and visualization techniques to compare perspectives on events related to the COVID-19 pandemic for the United States, the United Kingdom, Canada, and Australia, and investigated the impact of COVID-19 events from February to November 2020.

### Post Volume Variation for the COVID-19 Reddit Data Set

As shown in Figure 2, we observed that the post volume and user volume gradually decreased over the 10-month study period. We also observed that an early peak appeared during February and April 2020, which was the critical period for fighting the spread of COVID-19 in the United States, the United Kingdom, Canada, and Australia. One potential reason for the decline in post volume is that some users may avoid social media since they experienced increased anxiety from COVID-19–related news and discussions, and sought to protect their mental health [64]. Another reason is that users may become habituated to the “new normal,” which is identified as the acceptance phase after the authorities imposed social distancing measures [65]. In this stage, Aiello et al [65] found that people were more open to find solutions to continue social interaction; for example, the number of visits to parks and outdoor spaces increased. Hence, users posted less content in COVID-19–related subreddits to seek physical social support.

From the large volume of posts, we can see that Reddit supports the collection of a large volume of data that can provide insights into population attitudes and behavior. Previous studies have demonstrated that the analysis of public behavior and attitudes can help public health agencies and policymakers cope effectively in times of crisis [1].

### Topic Variation Among the United States, the United Kingdom, Canada, and Australia

The common topics shown in Table 2 varied among the four countries. As shown in Figure 3, we found that in the United States, the majority of the posts focused on COVID-19 prevention, with only a small portion of posts directly discussing COVID-19–related policies. For the United Kingdom, Canada, and Australia, the majority of posts focused on the impact of COVID-19, including job loss, food insecurity, and feelings of anxiety. Especially for the United Kingdom and Australia, users’ concerns—at least as expressed in these subreddits—focused on the impact of COVID-19 and government policies. At the beginning of the pandemic, a core concern among the Reddit**-**using population centered on effective COVID-19 prevention strategies due to the scientific uncertainties regarding how the virus was transmitted [9,10]. The social impact of COVID-19 is also a leading topic, which is consistent with the fact that the COVID-19 crisis poses huge psychological pressure and is associated with mental health issues [12-14].

As shown in Figure 4, we found that the totality of topic-related posts reached a peak in March when all four countries announced a lockdown and enforced travel restrictions (see Table 5 for a summary of lockdown events). Especially in March 2020, when the COVID-19 outbreak started and governments enforced border shutdowns, travel restrictions, and quarantine [5-7], people’s topics focused on the impact of COVID-19, including education and economic disruptions [8].

### Limitations

The work reported in this paper is not without limitations. COVID-19–related subreddits are still relatively new, with most of them initiated in February 2020. In the early stages of the COVID-19 pandemic, a considerable volume of COVID-19–related rumors spread [66] making Reddit data less reliable for the purposes of monitoring the outbreak, but useful for monitoring disinformation and public concerns. Additionally, Reddit has known sociodemographic biases. For example, the service is more popular in urban and suburban areas than in rural areas [67].

Topic modeling with LDA has a number of limitations, especially with respect to assessing topic quality. We noticed two problems when we manually checked the topic models: (1) very similar posts (eg, COVID-19 case report) may be assigned to different topics and (2) very simple posts (eg, lockdown announcement) may correlate to many topics. Similar problems were discovered by Xu et al [68] when analyzing clinical data.

Another issue in this work is related to the completeness of the Reddit data we collected via the pushshift.io API [52]. Although pushshift.io allows collecting a large amount of historical data from Reddit and yields a more complete data set than alternative methods (eg, the PRAW API) [53], it failed to identify all new updates, including deleted comments [54]. Even though we recollected the data to make it more complete, the Reddit data we curated may still be missing data.

A further limitation is related to the differences in culture associated with different subreddits. As Reddit data do not in general include geolocation information, we collected data from the six most popular COVID-19 subreddits related to the United States, the United Kingdom, Canada, and Australia. We examined the posts and noticed that most users are local people (ie, users from *r/CanadaCoronavirus* are mostly Canadians). Thus, the subreddits not only reflect people’s opinions but also the culture differences in the four countries. For example, people in the United Kingdom concentrate on discussing politics or COVID-19–related breaking news. Thus, the leading topic, politics-related policies, in *r/CoronavirusUK* does not fully reflect people’s concerns related to COVID-19, as it may simply reflect people’s discussion habit in the United Kingdom. Therefore, the differences in topics may not fully reflect people’s opinion toward COVID-19 in the United States, the United Kingdom, Canada, and Australia.

Finally, in this work we did not explicitly consider the demographic characteristics (eg, age, socioeconomic status, gender [52,69]) of Reddit users across the four countries and how these characteristics may differ.

### Conclusion

In this work, we used Reddit data to examine variations in people’s concerns during the COVID-19 crisis in the United States, the United Kingdom, Canada, and Australia. We found that people posted more on Reddit during lockdown events, and people’s concerns differ among the four countries. Further, this work provides evidence to support the contention that there are key differences between salient topics discussed across the four countries on the Reddit platform. Further, our approach indicates that Reddit data have the potential to provide insights not readily apparent in survey-based approaches.

## Appendix

Multimedia Appendix 1 Calculation for the topic bar chart in Figure 3.

Multimedia Appendix 2 Higher resolution version of Figure 4. Topic weekly trend for (A) the United States, (B) Australia, (C) Canada, and (D) the United Kingdom.

## Abbreviations

API: application programming interface
LDA: latent Dirichlet allocation
WHO: World Health Organization
