# Supplementary material for: Perspectives of the COVID-19 Pandemic on Reddit: Comparative Natural Language Processing Study of the United States, the United Kingdom, Canada, and Australia
Source: JMIR Infodemiology. 2022 Sep 27;2(2):e36941. doi: 10.2196/36941 (PMC9521381; doi:10.2196/36941)
Supplement: Multimedia Appendix 1 [file infodemiology_v2i2e36941_app1.docx]

Figure 3 Topic Bar Chart Calculation

|  | CAN | UK | AUS | US |
| --- | --- | --- | --- | --- |
| Total # posts | 14271 | 17307 | 16630 | 35768 |
| #Posts (ratio) in Case Report | 2264 (16%) | 210 (1.2%) | 3510 (21%) | 217 (0.6%) |
| #Posts (ratio) in COVID Impact | 11232 (79%) | 13791 (80%) | 7540 (45%) | 351 (1%) |
| #Posts (ratio) in Prevention | 727 (5%) | 7 (0.04%) | 2588 (16%) | 35085 (98%) |
| #Posts (ratio) in Policy and News | 48 (0.3%) | 3299 (19%) | 2992 (18%) | 115 (0.3%) |
