# Supplementary figures and images for: Perspectives of the COVID-19 Pandemic on Reddit: Comparative Natural Language Processing Study of the United States, the United Kingdom, Canada, and Australia
Source: JMIR Infodemiology. 2022 Sep 27;2(2):e36941. doi: 10.2196/36941 (PMC9521381; doi:10.2196/36941)

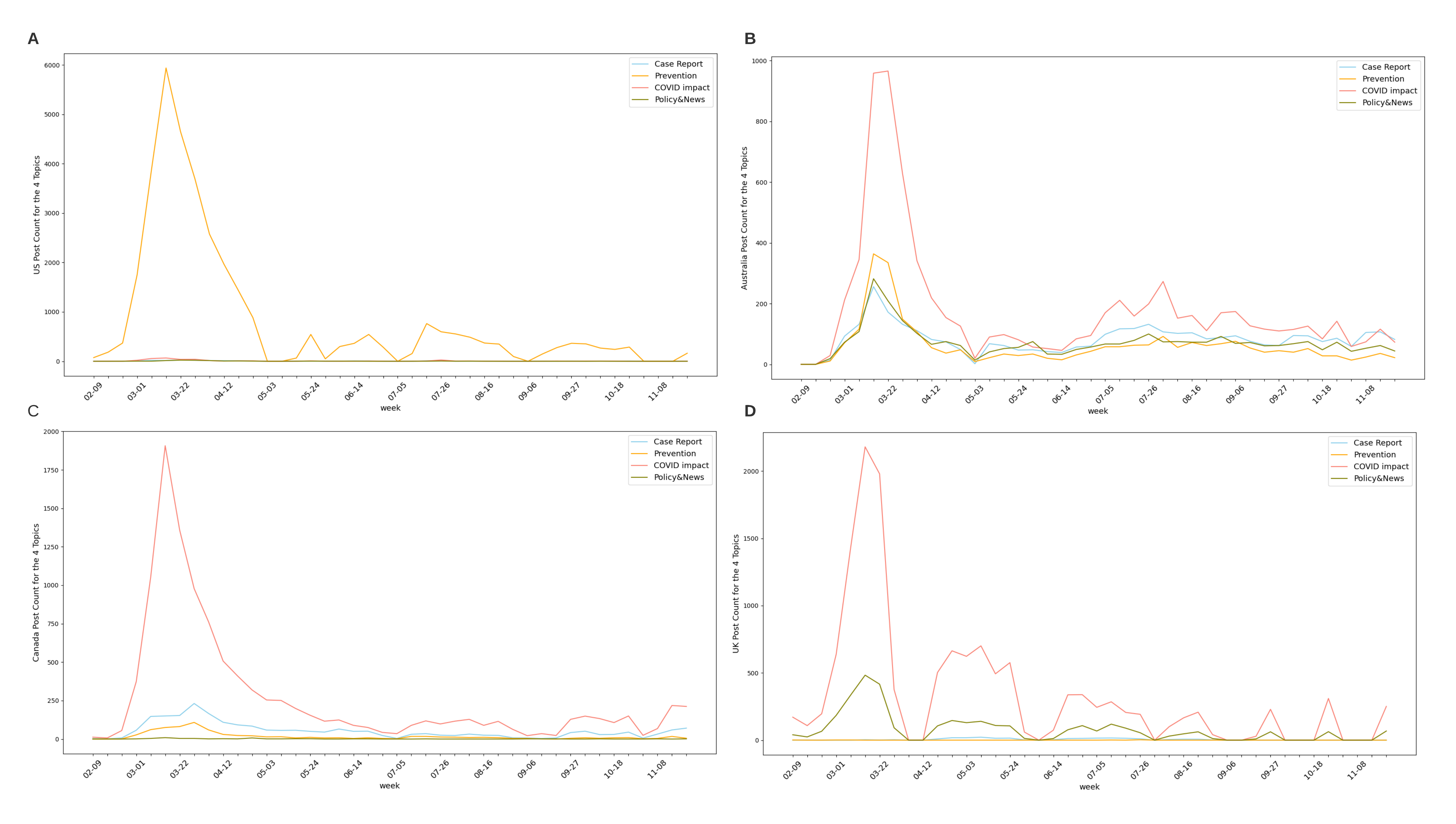

Supplement: Multimedia Appendix 2 [file infodemiology_v2i2e36941_app2.png]
